# A Static-Cidal Assay for *Trypanosoma brucei* to Aid Hit Prioritisation for Progression into Drug Discovery Programmes

**DOI:** 10.1371/journal.pntd.0001932

**Published:** 2012-11-29

**Authors:** Manu De Rycker, Sandra O'Neill, Dhananjay Joshi, Lorna Campbell, David W. Gray, Alan H. Fairlamb

**Affiliations:** Drug Discovery Unit, Division of Biological Chemistry and Drug Discovery, University of Dundee, Dundee, United Kingdom; University of Pittsburgh, United States of America

## Abstract

Human African Trypanosomiasis is a vector-borne disease of sub-Saharan Africa that causes significant morbidity and mortality. Current therapies have many drawbacks, and there is an urgent need for new, better medicines. Ideally such new treatments should be fast-acting cidal agents that cure the disease in as few doses as possible. Screening assays used for hit-discovery campaigns often do not distinguish cytocidal from cytostatic compounds and further detailed follow-up experiments are required. Such studies usually do not have the throughput required to test the large numbers of hits produced in a primary high-throughput screen. Here, we present a 384-well assay that is compatible with high-throughput screening and provides an initial indication of the cidal nature of a compound. The assay produces growth curves at ten compound concentrations by assessing trypanosome counts at 4, 24 and 48 hours after compound addition. A reduction in trypanosome counts over time is used as a marker for cidal activity. The lowest concentration at which cell killing is seen is a quantitative measure for the cidal activity of the compound. We show that the assay can identify compounds that have trypanostatic activity rather than cidal activity, and importantly, that results from primary high-throughput assays can overestimate the potency of compounds significantly. This is due to biphasic growth inhibition, which remains hidden at low starting cell densities and is revealed in our static-cidal assay. The assay presented here provides an important tool to follow-up hits from high-throughput screening campaigns and avoid progression of compounds that have poor prospects due to lack of cidal activity or overestimated potency.

## Author Summary

*Trypanosoma brucei* is a protozoan parasite causing African sleeping sickness. Current treatments for this disease have significant limitations, underlining the need for better and safer drugs. To identify new chemical starting points for drug development, large compound collections are screened against the parasite. Such screens typically do not distinguish between compounds that slow the growth of the parasite and compounds that actually kill the parasite (cidal compounds). Here, we present the development of an assay to identify such compounds. The main advantage of our assay is that it marries a relatively high-throughput to increased understanding of mode of action. Many active compounds (hits) are usually identified in *T. brucei* primary screening campaigns, making it difficult to select which compounds should undergo further development. Our assay allows testing of all of the hits for cidal activity so that only the most promising compounds are progressed. We show that the starting cell density used in the *T. brucei* growth assay can have a significant effect on the shape of dose response curves, and that important information regarding the mode of action of a compound can remain hidden at low starting densities as used commonly in *T. brucei* screening assays.

## Introduction

Human African Trypanosomiasis (HAT) or sleeping sickness is an endemic disease of sub-Saharan Africa. It is caused by two subspecies of *Trypanosoma brucei*, *T. b. rhodesiense* and *T. b. gambiense*, the latter of which is responsible for 95% of all cases of HAT [1]. Progress has been made in reducing the incidence of the disease and in 2009, for the first time in 50 years, the number of new reported cases has dropped below 10,000 [2]. Currently, the early-stage drug treatments are pentamidine and suramin for *T. b. gambiense* and *T. b. rhodesiense* respectively, whereas late stage disease (when the parasites have spread to the central nervous system) is treated with nifurtimox eflornithine combination therapy (NECT) for *T. b. gambiense* and the arsenic based compound melarsoprol for *T. b. rhodesiense*. In spite of the apparent success of the current treatments they are associated with a series of problems, including complexity of administration, adverse side-effects and the emergence of resistance [3]. Thus, it is clear that it is essential to continue the development of new drugs.

There are two main avenues to identify new chemical matter with anti-trypanosomal activity; target-based screening and phenotypic screening. Both methods have their own merits, and can complement each other [4], [5]. Whichever method is used, it is desirable to identify compounds with fast trypanocidal (killing of the parasites), rather than trypanostatic (inhibition of parasite growth) activity, as it is more likely that such compounds can be developed into fast acting drugs with improved treatment regimens compared to the current options. This is reflected in the current DNDi Target Product Profile for HAT which specifies that new candidate drugs should ideally be cidal [6]. Experiments determining the cidal versus static nature of compounds are routinely carried out in the antibacterial field [7], and have also been described for malaria [8] and *Trypanosoma cruzi* [9].

High-throughput phenotypic screens for *T. brucei* are often carried out using an endpoint growth assay with either a redox indicator [10], [11], [12], [13], [14] or ATP-dependent luminescence [15], [16] as a measure for trypanosome cell density. Typically the trypanosomes are grown in the presence of test compounds for several days with a starting cell-density well below the detection limit of the assay. This makes distinguishing compounds with a cidal effect from compounds with a static effect impossible. Follow-up experiments to demonstrate the cidal nature of a compound are relatively labour intensive and often fall outside the remit of hit discovery programmes as they are difficult to automate and not suitable for screening large amounts of compounds. In our own experience we have progressed apparently potent hits from a primary resazurin-based *T. brucei* screen all the way to *in vivo* efficacy studies where the series failed. Further studies showed that the minimum cidal concentration for these compounds was much higher than expected from our routine *T.brucei* assay. This led to the development of the high-throughput 384-well trypanosome growth assay presented here which provides an initial, quantitative indication of the cidal nature of anti-trypanosome compounds. We further show that the starting-density used in trypanosome growth assays can have a significant effect on the shape of potency curves, and that at low starting-density important information regarding the mode of action of a compound may be hidden.

## Methods

### Chemicals and materials

Melarsoprol was a gift from Rhone-Poulenc (France). Resazurin, pentamidine, nifurtimox, eflornithine (D,L-α-difluoromethylornithine; DFMO) and suramin were obtained from Sigma. White clear bottom plates for *T. brucei* growth assays were obtained from Greiner and echo plates were from LabCyte.

### Trypanosome culture

Bloodstream-form *T. b. brucei* (strain 427, ‘single marker’ cloned line) was grown at 37°C in presence of 5% CO_2_ in HMI9-T medium [10]. For routine culturing cells were diluted to 2×10^4^ ml^−1^ for 48-h passages or diluted to 2×10^3^ ml^−1^ for 72-h passages. Cells were maintained in vented T75 flasks. Cultures used for screening were split to 2×10^5^ ml^−1^ the day before plating in 384-well plates.

### Limit of detection

*T.brucei* cells were counted and dispensed into a 384-well plate at the following densities: 0, 5×10^3^, 1×10^4^, 2×10^4^, 5×10^4^, 1×10^5^, 2×10^5^, 5×10^5^, 1×10^6^ and 2×10^6^ cells ml^−1^. Each cell density was plated into 16 wells. Resazurin was then added at 0.05 mM final concentration and the plates were incubated for 4 h at 37°C. Fluorescence intensity was then measured using a Perkin Elmer Victor 3 plate-reader (excitation 528 nm, emission 590 nm). The limit of detection was calculated as the number of cells giving a signal greater than the mean signal of the blank wells plus 3 times the standard deviation of the blank wells.

### Compound preparation

For the preparation of potency curves: 30 µl of compound at 10 mM in DMSO was manually dispensed into 384-well compound-holding plates. A ten-point three-fold dilution curve in DMSO was then created on a Perkin Elmer Janus liquid handling robot. For the preparation of assay plates, serial dilution curves were transferred to LabCyte Echo certified plates, and 250 nl of each concentration was dispensed into white clear bottom 384-well assay plates using a LabCyte Echo acoustic dispenser. For static-cidal assays three replicate assay plates were created, one for each time point. Columns 11, 12, 23 and 24 of each plate contained DMSO only. The final concentration of DMSO in all assay wells was 0.5% (v/v).

### Growth assays

Standard growth assays were carried out as described previously [17]. In short, bloodstream-form *T. brucei* cells in fresh medium were plated into columns 1–22 of 384-well assay plates using a Wellmate dispenser (Thermo Fisher) (5×10^3^ ml^−1^, 50 µl/well) and incubated for 68 h at 37C, 5% CO_2_. Next resazurin was added at 0.05 mM final concentration and the plates were incubated for a further 4 h. Fluorescence was then measured using a Perkin Elmer Victor 3 plate-reader (excitation 528 nm, emission 590 nm). For the static-cidal assay 3 replicate assay plates were prepared and trypanosomes were seeded into each plate at 4×10^5^ ml^−1^ (50 µl/well), unless otherwise indicated. Immediately thereafter resazurin was added to one of the plates, and all plates were incubated at 37°C, 5%CO_2_. After 4 h the time = 0 plate was read as above. Twenty hours later the second plate was processed in the same way, and at 44 h the last plate was processed. Z-factors were calculated for each plate using the following formula: 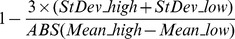. A minimum z-factor of 0.6 was set as quality cut-off. Growth rates were determined for both assays by harvesting cells every 24 hours from 384-well plates and counting them in a CASY Counter (Roche). This was carried out on three occasions and growth curves were plotted.

### Data analysis

Dose-response curve fitting was carried out either in IDBS ActivityBase or using the Excel Add-in XL-fit (IDBS). For monophasic fits the following 4 parametric equation was used:
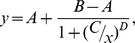
where A = % inhibition at bottom, B = % inhibition at top, C = EC_50_, D = slope, *x* = inhibitor concentration and y = % inhibition. For biphasic fits equation: 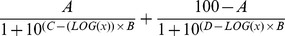 was used, with A = % inhibition at mid-plateau, B = slope, C = log(EC_50_^A^), D = log(EC_50_^B^)). Inhibition at the bottom of the curve is fixed to 0% and at the top to 100%.

Potency values are given as pEC_50_ (the negative logarithm of the EC_50_ in molar units). All data are the result of at least 3 independent measurements, reported as arithmetic mean of pEC_50_ values. Growth curves for the static-cidal assay were produced in Microsoft Excel by plotting the raw resorufin fluorescence measurement for each drug concentration against time. The average of three independent measurements was calculated and plotted together with the standard deviation. The Minimum Cidal Concentration (MCC) was defined as the lowest concentration of drug that results in a decrease of resorufin signal over time. For compound concentrations that allowed significant growth during the 0–24-h window (resorufin RFU>10 at 24 h) followed by a decrease in signal during the 24–48-h window, we set the additional requirement that the signal at 48 h had to be equal to, or less than the starting signal (t = 0 h) to account for the noise in the assay at the 48-h time point and to assure that cidal action was indeed occurring.

## Results

**Figure 1:**
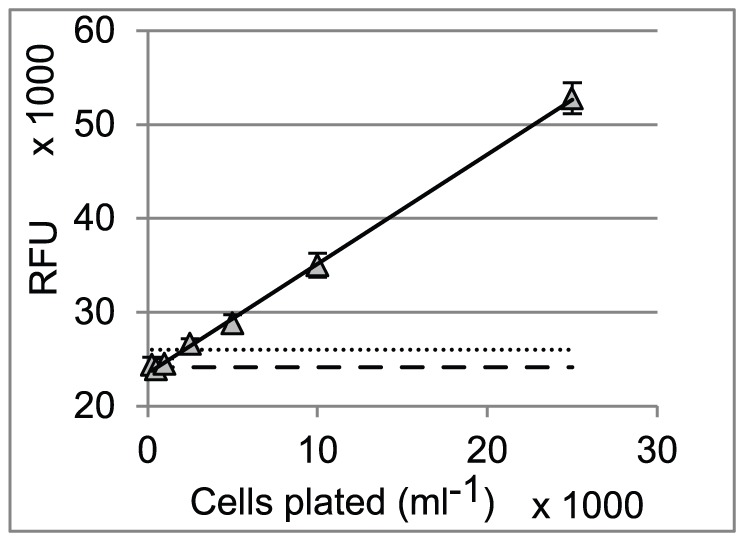
Detection limit in *T. brucei* static-cidal screening assay. Solid line shows linear regression (R^2^ = 0.999) for cell number versus fluorescence signal (RFU). Dashed line shows average value of blanks. Dotted line shows detection limit for the assay (5×10^4^ ml^−1^), calculated as 3 times the standard deviation of the blank.

**Figure 2:**
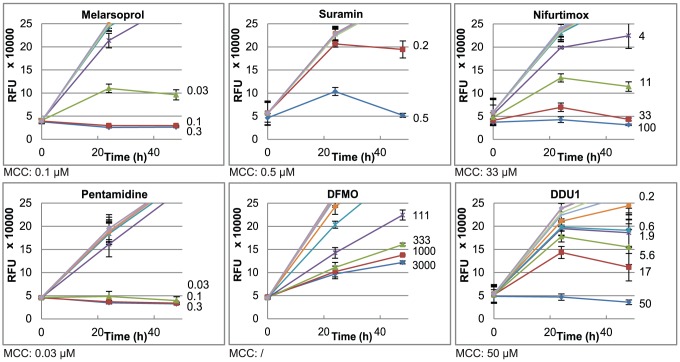
Growth curves obtained in the static-cidal assay for control compounds and representative test compound. Growth curves are shown for each compound, with the concentration of compound indicated on the right hand side of each growth-line (µM). MCCs are indicated for each compound. All measurements are the average of three independent experiments. The error bars indicate the standard deviation. RFU = relative fluorescence units.

The routine *T. brucei* screening assay employed in our unit involves plating the parasites at a density of 250 cells per well (in 50 µl) into 384-well plates with pre-dispensed compounds. After 68 h of incubation the live-cell indicator resazurin is added and incubated for 4 h followed by measuring resorufin fluorescence in a plate-reader. As this starting-density is below the detection limit of the assay (2,500 cells/well, dotted line in Figure 1), it is impossible to distinguish cidal compounds from static compounds, or even compounds that effect a moderate level of growth inhibition. To obtain a measurement of the cidal nature of compounds we altered our standard assay to allow the monitoring of cell growth from the moment of addition of the trypanosomes to the compounds. We increased the starting-density 80-fold (2×10^4^ cells per well), well above the detection limit of the assay, which allowed us to use the resazurin method immediately after mixing parasites with compounds to obtain a time zero measurement of cell density. We then repeated the resazurin measurement at 24 h and 48 h to obtain a growth curve (later time points could not be measured as the cells cease growing due to nutrient depletion or acidification of the media). After the final time point, growth curves were created for each concentration of compound. A panel of known inhibitors of *T. brucei* growth were tested in this assay (Figure 2). A decline in signal over time indicates a reduction in the number of trypanosomes and thus cidal activity. A quantitative measurement of the lethality of a compound is given by the MCC, the lowest concentration at which a decline in signal is seen during the 48-h drug exposure (see methods). Cidal activity was observed for melarsoprol (MCC 0.1 µM), suramin (MCC 0.5 µM), nifurtimox (MCC 33 µM) and pentamidine (MCC 0.03 µM). DFMO only had a growth reducing effect. A series of compounds (unpublished) from our HAT drug discovery programme were tested in this assay and the growth curves for a representative compound is also shown (DDU1; MCC 50 µM). The structure for DDU1 is given in supplementary figure 1.

We next compared the potencies obtained in our standard growth assay with the MCCs from the static-cidal assay. As shown in Table 1, the fold change between the two measures was markedly larger for compound DDU1 compared to the cidal control compounds, but similar to the cytostatic drug DFMO.

**Table 1:** Comparison of potency in the standard assay with MCC from the static-cidal assay.

Compound	pEC50 (std)	pMCC*	Fold change
Melarsoprol	8.05±0.01	7.0	11
Pentamidine	8.57±0.02	7.5	12
Suramin	6.74±0.04	6.3	3
Nifurtimox	5.45±0.01	4.5	9
DFMO	4.70±0.02	<2.5	>160
DDU1	6.75±0.06	4.3	283

* pMCC is calculated as −log(MCC) where concentration is in molar terms.

**Figure 3:**
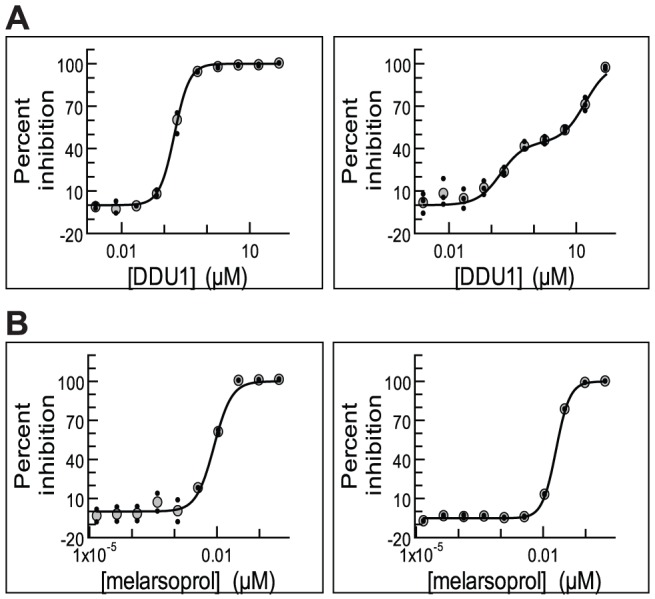
Effect of the assay protocol on the dose-response profile. A: In the standard assay (left) compound DDU1 displays monophasic behaviour, while a biphasic fit is obtained in the static-cidal assay (right). B: Melarsoprol shows monophasic behaviour in both the standard assay (left) and the static-cidal assay (right).

**Figure 4:**
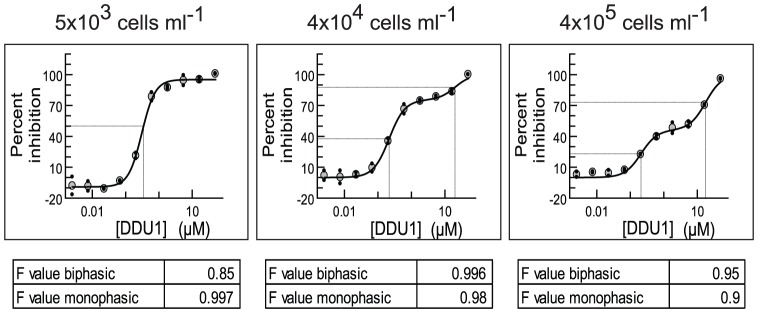
Starting density affects the position of the mid-plateau for compounds with biphasic behaviour. *T. brucei* were plated at 5×10^3^ ml^−1^ (left), 4×10^4^ ml^−1^ (middle) and 4×10^5^ ml^−1^ (right) in 384-well plates and grown for 48 h in presence of 10 different concentrations of compound DDU1. Potency curves were plotted, using both a mono-phasic and biphasic equation and the best fit (as determined by the F-value) is shown (biphasic for 4×10^5^ ml^−1^ and 4×10^4^ ml^−1^; monophasic for 5×10^3^ ml^−1^).

**Figure 5:**
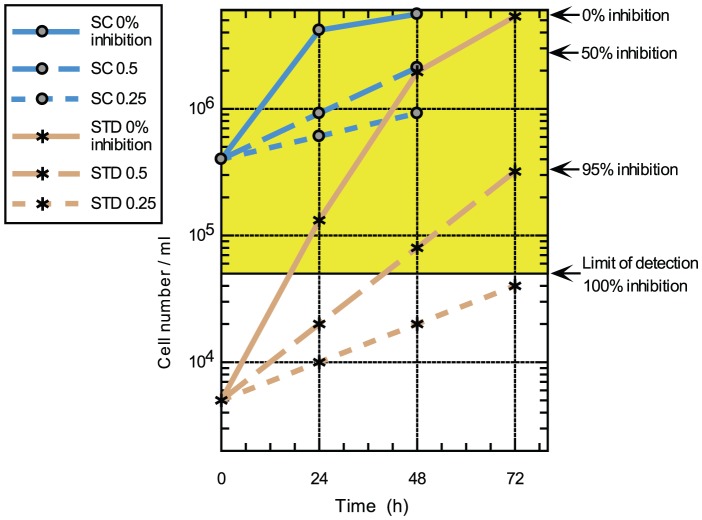
Model to explain the effect of starting-density on the dose-response profile. Measured (solid lines, 0% inhibition, growth rate = 1) and predicted (dashed lines) growth curves for *T. brucei* in the standard (STD) and static-cidal (SC) assays. Predicted growth curves are for 50% inhibition of growth rate (long dashes, growth rate = 0.5) and 75% inhibition of growth rate (short dashes, growth rate = 0.25). Percent inhibition as calculated at t = 72 h (STD) or t = 48 h (SC) is given on right hand side. The yellow shaded area represents cell densities above the detection limit.

**Figure 6:**
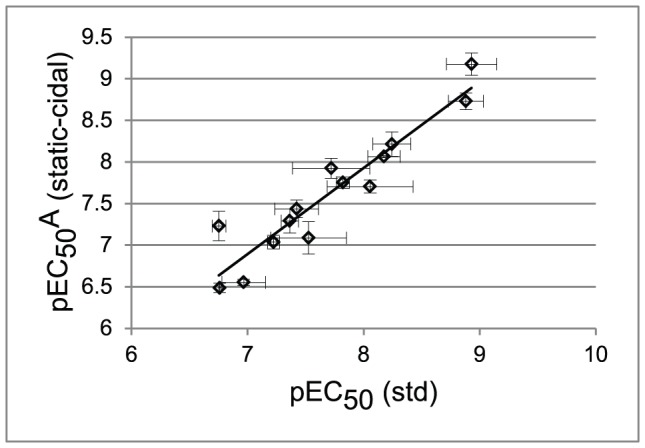
Correlation between pEC_50_ (std) and pEC_50_^A^ (static-cidal). The pEC_50_^A^ from the static-cidal assay for compounds that show biphasic behaviour is plotted as a function of pEC_50_ obtained in the standard monophasic assay. All results are from at least three independent replicates. Dashed line shows linear regression fit (R^2^ = 0.89).

To investigate this further we fitted potency curves using the data obtained in the static-cidal assay at the 48 h time point, and compared these to potency curves obtained in the standard assay. The data for the standard assay were obtained at 48 h and 72 h, to assess any effects of exposure time, and no significant differences were observed (data not shown). Figure 3A shows the dose-response curves for compound DDU1.The data obtained in the standard assay fitted best to a monophasic dose-response curve (left panel) while the data from the static-cidal assay fitted much better to a biphasic curve (right panel). Melarsoprol, which showed a small difference between pEC_50_ (std) and pMCC exhibited monophasic curves under both conditions (Figure 3B). The main difference between the two assays is the starting cell-density; we thus investigated whether the appearance of the biphasic dose-response curve was cell-density dependent. Figure 4 shows potency curves obtained for DDU1 with three different starting-densities (4×10^5^, 4×10^4^ and 5×10^3^ ml^−1^). As expected, the data fitted a monophasic model at 5×10^3^ ml^−1^. Biphasic behaviour was observed for both 4×10^4^ and 4×10^5^ ml^−1^ starting-densities, but interestingly the mid-plateau was positioned much higher at the lower of the two densities (∼80% compared to ∼50%). These data confirm that the starting-density can have a major effect on the shape of dose-response curves. The explanation for this behaviour lies in the effect of the assay detection limit on the end-point measurements. In the standard assay, which starts at a cell-density well below the assay detection limit, the cell-density in a well exposed to a compound that inhibits growth by 50%, i.e. a twofold increase in the doubling time, will show 95% inhibition compared to untreated control (Figure 5). Potency curves will thus show near complete inhibition for a concentration of compound that only moderately inhibits cell growth. This problem is resolved by increasing the starting-density to above the detection limit as done in our static-cidal assay, and as a result any level of inhibition can now be detected. Reducing the starting density will result in increasing overestimation of compound activity, which explains why the mid-plateau seen in Figure 4 moves up with decreasing starting-density. Following from this, EC_50_^A^ of the biphasic curves should match the EC_50_ obtained from the monophasic curves using the low starting-density. Figure 6 shows this correlation for the set of compounds with biphasic behaviour in the static-cidal assay, and indeed good correlation (R^2^ = 0.9) is seen between pEC_50_^A^ (static-cidal) and pEC_50_ (std). Taken together these data show that the starting-density used in the assay has a major impact on the resulting potency curves, and, when too low, that important biologically relevant characteristics of compounds like the biphasic behaviour shown here may be hidden.

## Discussion

Here we describe a straightforward 384-well *T. brucei* growth assay that estimates the extent to which a compound is cidal. The quantitative MCC can be used to compare compounds and should be an essential component of the decision-making process for progression of a compound. Important advantages of this assay over existing procedures are the high-throughput that can be achieved and the straight-forward implementation of the assay as it is similar to the standard 384-well high-throughput assay [14] and does not require any additional resources. We routinely screen upwards of twenty 384-well plates in a single batch, which allows the static-cidal testing of nearly 200 compounds. We do not see this assay as a platform for primary screening; instead it is more suitable as a secondary assay to follow up hits from high-throughput screening campaigns run with the standard assay. As such screens can yield large numbers of hits, the throughput achieved in our static-cidal assay is essential to allow an assessment of the cidal nature of these hits. The MCC, together with other relevant criteria, can then be used to progress the most promising compounds into the hit-to-lead phase. On the down-side, the assay does not provide unequivocal proof of cidal activity and, to obtain this, more detailed wash-out experiments are required. Such studies can be carried out at a later stage in development for the most promising compounds.

We investigated the cidal activity of commonly used HAT drugs using our assay. As expected, we saw cidal activity for melarsoprol, suramin, pentamidine and nifurtimox. The known trypanostatic effect of DFMO was confirmed in our assay as we only detected a growth-retarding effect for this compound [18], [19]. A series of factors need to be taken into consideration when interpreting the results provided by the assay. The mode of action of the compound, concentration range tested, duration of exposure, compound stability, and cell density may all affect the growth-inhibition curves and the resulting MCCs. Compounds that act as polypharmacological agents may be static at lower concentrations when only one of the targets is inhibited, whereas at higher concentration they may be cidal due to the inhibition of other targets. Similarly, specific inhibition of a target may result in static behaviour, whereas cidal behaviour at higher concentrations may be the result of non-specific toxicity. The high cell density used in our static-cidal assay may affect the final intra-cellular concentration of the drug, in particular for compounds like pentamidine that are actively concentrated by the parasite [20], [21]. So, while extensive studies are required to properly characterise the cidal nature of a compound, our assay provides a powerful tool, using a set of standard conditions, to rank large sets of compounds and to aid in choosing the right candidate compounds for progression in a drug development programme.

We employed our assay to test a series of compounds, represented here by DDU1, which while appearing potent in the standard assay, and in spite of good ADME properties, did not show any *in vivo* efficacy (data not shown). The results revealed that there was a much larger fold-difference between the EC50 and MCC for this series (>200 fold) compared to the cidal control compounds (≤12 fold) (Table 1). This striking disparity made us investigate the differences between the standard assay and our static-cidal assay and led to the observation that obtaining potency curves using starting-densities below the detection limit may hide important characteristics of compounds relevant to their mode of action. In our case, the biphasic nature of one of our compound series remained hidden using our routine assay. This resulted in a severe overestimation of compound potency and progression of these compounds into *in vivo* efficacy studies, wasting a significant amount of resource. The main explanation for this behaviour is that moderate levels of growth inhibition may result in cell-densities below or close to the detection limit at the end of the assay, suggesting complete inhibition of cell growth. The implication is that standard resazurin-based *T.brucei* assays with low starting density do not actually report trypanosome viability; instead they show growth inhibition to the extent that the detection limit of the assay is not reached. By increasing the starting density substantially above the detection limit moderate levels of inhibition can be detected and in our case reveal the biphasic nature of the compound series in question. This biphasic behaviour is likely indicative of the compounds exerting their effect through at least two modes of action. Differential susceptibility in the cell population could be another explanation; however the fact that we use a clonal cell line in this study, and the observation that the position of the biphasic mid-plateau is cell-density dependent rule this out. An important consideration is the effect of starting density on the rate of cell growth across the time course. As can be seen on Figure 5, cell growth remains close to exponential for a 3-day time course when the low starting-density of 5×10^3^ ml^−1^ is used. However, with the high starting-density (4×10^5^ ml^−1^) cells start reaching stationary phase within 48 h. This should be avoided as it affects the accuracy of the percent inhibition calculations. This highlights the challenge when choosing drug-exposure time and starting density for screening fast growing organisms such as *T. brucei*, as these parameters can have a significant effect on apparent drug action (fast versus slow acting, monophasic versus biphasic behaviour, static versus cidal action). There is no one set of parameters that can be universally applied and suitable conditions need to be chosen depending on the questions being addressed. The effect of starting cell-density described here is likely not unique to trypanosomes and may apply to growth assays used in, amongst others, the anti-bacterial and anti-cancer fields.

## Supporting Information

Figure S1 Chemical structure of compound DDU1.
(EPS)
